# Records of Limoniidae and Pediciidae (Diptera) from Armenia, with the first Armenian checklist of these families

**DOI:** 10.3897/zookeys.585.8330

**Published:** 2016-04-27

**Authors:** Jozef Oboňa, Jaroslav Starý, Peter Manko, Ľuboš Hrivniak, Levon Papyan

**Affiliations:** 1 Department of Ecology, Faculty of Humanities and Natural Sciences, University of Prešov, 17. novembra 1, SK–081 16 Prešov, Slovakia; 2 Neklanova 7, CZ–779 00 Olomouc-Nedvězí & Silesian Museum, Nádražní okruh 31, CZ–746 01 Opava, Czech Republic; 3 Biology Centre CAS, Institute of Entomology, Branišovská 1160/31, CZ–370 05 České Budějovice, Czech Republic; 4 Faculty of Sciences, University of South Bohemia, Branišovská 31, CZ–370 05 České Budějovice, Czech Republic; 5 Scientific Center of Zoology and Hydroecology, Institute of Zoology, 7, Sevak Str., Yerevan 0014, Republic of Armenia

**Keywords:** Diptera, Limoniidae, Pediciidae, distributions, first records, checklist, Armenia

## Abstract

Records of species of the families Limoniidae and Pediciidae are presented from Armenia. A total of 38 species of Limoniidae and four species of Pediciidae are listed. Of these, 27 species of Limoniidae and one species of Pediciidae represent the first records from Armenia. The first checklist of these families from Armenia is appended, containing 77 species of Limoniidae and six species of Pediciidae.

## Introduction

Compared to some European countries, Transcaucasia (Georgia, Armenia, Azerbaijan) is among less-investigated territories, as far as the families Limoniidae and Pediciidae are concerned. The territory occupies the southern macro-slopes of the Great Caucasus and the mountains and plateaux as far south as the Turkish and Iranian borders. Savchenko (e.g. 1971, 1972a,b, 1973, 1974, 1976a,b, 1978a,b, 1979, 1981, 1983, and others) contributed considerably to the knowledge of the local fauna and summarized his results in a comprehensive study (Savchenko 1989) dealing with the fauna of the former USSR. Faunal records from all relevant publications are registered in the Catalogue of the Craneflies of the World (Oosterbroek 2015). A total of 50 species of Limoniidae and five species of Pediciidae have been previously listed from Armenia.

The material we present in this paper was mostly collected in the north-western part of Armenia during a recent sampling campaign from August 26 to September 4, 2015. Thirty-eight species of Limoniidae and four species of Pediciidae were identified from this material, of which 27 species of Limoniidae and one species of Pediciidae represent the first records from Armenia. Seven species of Limoniidae and one species of Pediciidae are new to the whole Transcaucasia. In addition, we append the first checklist of Limoniidae and Pediciidae from Armenia, containing 77 species of Limoniidae and six species of Pediciidae.

## Material and methods

**Map 1.**
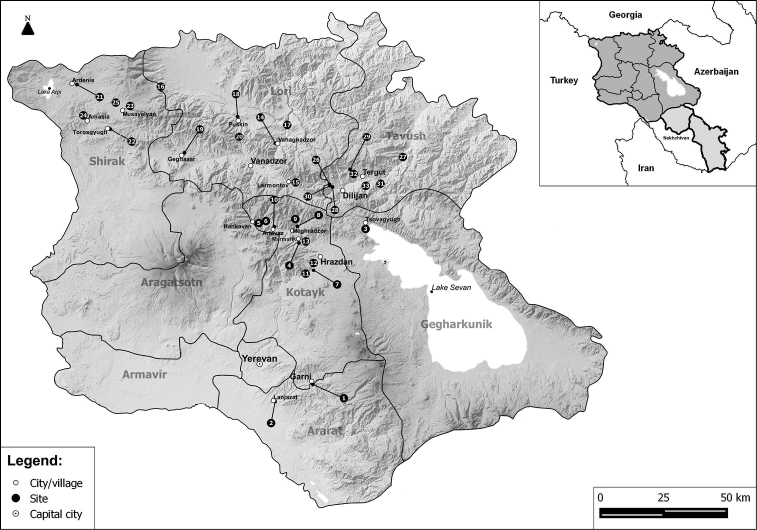
Map showing all sampling sites in Armenia.

Samples were collected by sweep-netting from vegetation along streams and lakes by the first, third, and fourth authors (JO, PM, LH) and preserved in 75% ethanol. A list of 33 sampling sites, with coordinates and altitudes, is given in Table 1, and the locations of the sites are shown in Map 1. The material is deposited in the collection of the second author (JS) who also identified the species. Some specimens were dried and mounted on points in the course of the study. The male terminalia, if necessary, were prepared by boiling in a solution of 10% KOH and preserved in glycerine in a sealed plastic tube pinned with the appropriate specimen after examination. Classification, nomenclature, and distribution for individual species are given as summarized by Oosterbroek (2015).

**Figures 1–3.**
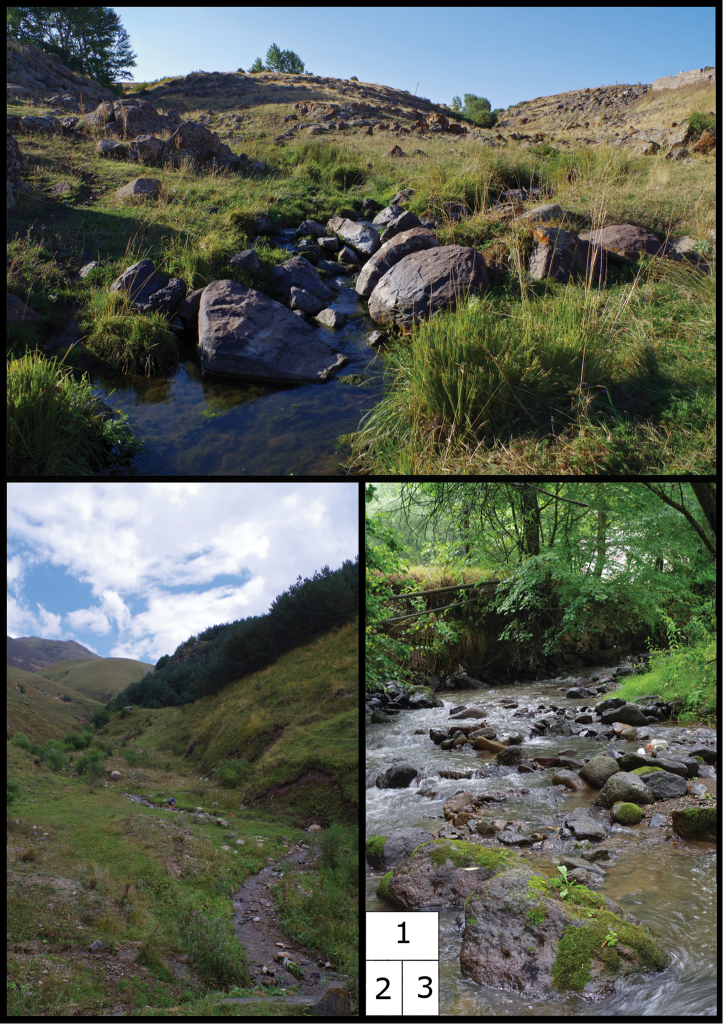
Sampling sites with highest species diversity. **1** Site No. 24 - NW of Amasia, tributary of Akhurian River (9 species, including *Erioconopa symplectoides*, Molophilus (Molophilus) pleuralis, Ormosia (Ormosia) hederae,﻿ and *Limonia macrostigma*) **2** Site No. 15 - Lermontov, tributary of Aghstev River (8 species, including Hoplolabis (Parilisia) iranica, Ormosia (Ormosia) hederae, Rhabdomastix (Rhabdomastix) filata, Dicranomyia (Dicranomyia) melanantha, *Limonia macrostigma*, ﻿and *Limonia stigma*) **3** Site No. 28 - W of Dilijan, Bldan River (8 species, including Pseudolimnophila (Pseudolimnophila) melanura, Antocha (Antocha) vitripennis, Dicranomyia (Dicranomyia) pontica, ﻿and *Limonia hercegovinae*).

**Table 1.** List of sampling sites.

Site No.	site name (province, short description of locality)	latitude/ N	longitude/ E	altitude/ m a.s.l.
1	Ararat Province: Garni, below Garni Temple, Azat River	40°06'39.4"	44°43'45.3"	1273
2	Ararat Province: nr. Lanjazat, Azat River	40°03'27.0"	44°34'38.3"	976
3	Gegharkunik Province: Tsovagyugh, nr. Sevan Lake	40°37'01.8"	44°57'44.2"	1930
4	Kotayk Province: between Marmarik and Aghavnadzor, tributary of Marmarik River	40°33'52.0"	44°40'09.1"	1872
5	Kotayk Province: E of Hankavan, Marmarik River	40°38'04.7"	44°29'19.4"	1974
6	Kotayk Province: E of Hankavan, Marmarik River	40°38'09.2"	44°32'23.2"	1913
7	Kotayk Province: Hrazdan Sewage Treatment Plant, Hrazdan River	40°29'12.8"	44°43'55.9"	1705
8	Kotayk Province: Meghradzor, behind railway, tributary of Marmarik River	40°37'12.7"	44°40'18.3"	1825
9	Kotayk Province: Meghradzor, below “Gold Mine”, tributary of Marmarik River	40°37'53.0"	44°40'17.5"	1870
10	Kotayk Province: near Artavaz, Marmarik River	40°36'49.9"	44°34'18.2"	1849
11	Kotayk Province: N of Solak, Hrazdan River	40°28'19.7"	44°42'42.2"	1567
12	Kotayk Province: SW of Hrazdan Reservoir, tributary of Hrazdan River	40°30'10.2"	44°44'22.4"	1718
13	Kotayk Province: between Marmarik and Aghavnadzor, Marmarik River	40°34'29.3"	44°41'02.2"	1760
14	Lori Province: W of Vahagnadzor, Zamanlu River	40°53'07.0"	44°34'39.0"	1092
15	Lori Province: Lermontov, tributary of Aghstev River (Fig. 2)	40°45'24.6"	44°38'42.0"	1853
16	Lori Province: Meghvahovit, road H31, small steppe brook	41°03'59.9"	44°05'44.2"	1949
17	Lori Province: N of Dzoraget, tributary of Pambak River	40°56'52.7"	44°37'37.2"	1030
18	Lori Province: N of Pushkin, tributary of Dzoraget River	40°58'04.8"	44°24'49.7"	1485
19	Lori Province: NE of Geghasar, Pambak River	40°51'17.4"	44°11'40.8"	1627
20	Lori Province: road H23 to Pushkin Pass, small brook	40°54'22.9"	44°25'33.3"	1839
21	Shirak Province: between Aghvoik and Ardenis, tributary of Akhurian River	41°04'21.2"	43°44'44.8"	2052
22	Shirak Province: E of Torosgyugh, tributary of Akhurian River	40°55'55.0"	43°52'45.3"	1885
23	Shirak Province: NE of Musayelyan, tributary of Akhurian River	41°00'13.2"	43°57'24.9"	2195
24	Shirak Province: NW of Amasia, tributary of Akhurian River (Fig. 1)	40°58'20.5"	43°46'06.9"	1987
25	Shirak Province: Zuygaghbyur, meanders of tributary of Akhurien River	41°00'56.1"	43°54'18.8"	2034
26	Tavush Province: below Jukhtakvank Monastery	40°45'11.8"	44°48'25.7"	1411
27	Tavush Province: car park on road M4, tributary of Aghstev River	40°50'32.0"	45°06'56.8"	760
28	Tavush Province: W of Dilijan, Bldan River (Fig. 3)	40°44'49.1"	44°49'03.5"	1354
29	Tavush Province: E of Haghartsin, tributary of Aghstev River	40°48'09.3"	44°53'43.7"	1382
30	Tavush Province: E of Matosavank Monastery	40°44'59.6"	44°48'29.2"	1392
31	Tavush Province: N of Gosh, Getik River	40°45'16.5"	45°01'18.4"	940
32	Tavush Province: NW of Teghut, tributary of Aghstev River	40°47'15.2"	44°54'58.0"	1197
33	Tavush Province: vicinity of Parz Lake	40°44'57.7"	44°57'33.3"	1376

## Results

### Faunistic records

#### Family Limoniidae

##### Subfamily Limnophilinae

###### Paradelphomyia (Oxyrhiza) brevifurca

Taxon classificationAnimaliaDipteraLimoniidae

Savchenko, 1976

####### Material examined.

Tavush: below Jukhtakvank Monastery, (site 26), 29.viii.2015, 1 ♂.

####### Distribution.

So far only known from North Caucasus and Georgia. First record from Armenia.

###### Paradelphomyia (Oxyrhiza) fuscula

Taxon classificationAnimaliaDipteraLimoniidae

(Loew, 1873)

####### Material examined.

Kotayk: between Marmarik and Aghavnadzor, tributary of Marmarik R. (site 4), 27.viii.2015, 1 ♂.

####### Distribution.

Europe; Iran. First record from Armenia and Transcaucasia.

###### Paradelphomyia (Oxyrhiza) senilis

Taxon classificationAnimaliaDipteraLimoniidae

(Haliday, 1833)

####### Material examined.

Lori: N of Dzoraget, tributary of Pambak R. (site 17), 1.ix.2015, 1 ♂.

####### Distribution.

Europe; Azerbaijan, Turkey; Kirghizia. First record from Armenia.

###### Phylidorea (Phylidorea) ferruginea

Taxon classificationAnimaliaDipteraLimoniidae

(Meigen, 1818)

####### Material examined.

Kotayk: Hrazdan Sewage Treatment Plant, Hrazdan R. (site 7), 27.viii.2015, 1 ♂ 3 ♀.

####### Distribution.

Europe; Azerbaijan, Turkey, Israel; Central Asia, Mongolia; West Siberia. First record from Armenia.

###### Pseudolimnophila (Pseudolimnophila) melanura

Taxon classificationAnimaliaDipteraLimoniidae

Savchenko, 1984

####### Material examined.

Tavush: W of Dilijan, Bldan R. (site 28), 28.viii.2015, 6 ♂ 3 ♀.

####### Distribution.

So far only known from Tajikistan. First record from Armenia and Transcaucasia; first record since original description.

####### Remark.

Due to syntopic occurrence in Armenia of Pseudolimnophila (Pseudolimnophila) melanura and Pseudolimnophila (Pseudolimnophila) sepium the former cannot be considered a subspecies of the latter, as suggested by Savchenko et al. (1992).

###### Pseudolimnophila (Pseudolimnophila) sepium

Taxon classificationAnimaliaDipteraLimoniidae

(Verrall, 1886)

####### Material examined.

Tavush: W of Dilijan, Bldan R. (site 28), 28.viii.2015, 1 ♂.

####### Distribution.

Europe; Morocco; Georgia, Azerbaijan, Turkey; Central Asia. First record from Armenia.

##### Subfamily Chioneinae

###### Erioconopa symplectoides

Taxon classificationAnimaliaDipteraLimoniidae

(Kuntze, 1914)

####### Material examined.

Kotayk: Hrazdan Sewage Treatment Plant, Hrazdan R. (site 7), 27.viii.2015, 1 ♂; Shirak: NE of Musayelyan, tributary of Akhurian R. (site 23), 2.ix.2015, 2 ♀; Shirak: Zuygaghbyur, meanders of tributary of Akhurien R. (site 25), 2.ix.2015, 3 ♂ 1 ♀; Shirak: NW of Amasia, tributary of Akhurian R. (site 24), 2.ix.2015, 5 ♂; Shirak: between Aghvoik and Ardenis, tributary of Akhurian R. (site 21), 3.ix.2015, 1 ♂ 1 ♀; Shirak: E of Torosgyugh, tributary of Akhurian R. (site 22), 3.ix.2015, 4 ♂ 1 ♀; Lori: NE of Geghasar, Pambak R. (site 19), 3.ix.2015, 2 ♂ 1 ♀.

####### Distribution.

Europe, except for northern countries; Morocco; Georgia, Azerbaijan, Turkey. First records from Armenia.

###### Erioptera (Erioptera) fusculenta

Taxon classificationAnimaliaDipteraLimoniidae

Edwards, 1938

####### Material examined.

Kotayk: NW of Artavaz, Marmarik R. (site 6), 26.viii.2015, 3 ♂ 1 ♀; Kotayk: Hrazdan Sewage Treatment Plant, Hrazdan R. (site 7), 27.viii.2015, 5 ♂; Kotayk: SW of Hrazdan Reservoir, tributary of Hrazdan R. (site 12), 27.viii.2015, 1 ♂; Kotayk: near Artavaz, Marmarik R. (site 10), 27.viii.2015, 1 ♀; Kotayk: Meghradzor, behind railway, tributary of Marmarik R. (site 8), 27.viii.2015, 1 ♂; Tavush: W of Dilijan, Bldan R. (site 28), 28.viii.2015, 3 ♂; Ararat: Garni, below Garni Temple, Azat R. (site 1), 31.viii.2015, 5 ♂; Lori: Meghvahovit, road H31, small steppe brook (site 16), 2.ix.2015, 1 ♀; Lori: N of Gosh, Getik R. (site 31), 4.ix.2015, 6 ♂ 5 ♀; Shirak: NE of Musayelyan, tributary of Akhurian R. (site 23), 2.ix.2015, 5 ♂ 2 ♀; Shirak: NW of Amasia, tributary of Akhurian R. (site 24), 2.ix.2015, 8 ♂ 2 ♀; Shirak: between Aghvoik and Ardenis, tributary of Akhurian R. (site 21), 3.ix.2015, 1 ♀; Shirak: E of Torosgyugh, tributary of Akhurian R. (site 22), 3.ix.2015, 2 ♂.

####### Distribution.

Europe; Georgia, Armenia, Azerbaijan, Turkey, Israel; Turkmenia.

###### Erioptera (Erioptera) lutea

Taxon classificationAnimaliaDipteraLimoniidae

Meigen, 1804

####### Material examined.

Kotayk: NW of Artavaz, Marmarik R. (site 6), 26.viii.2015, 1 ♂; Kotayk: between Marmarik and Aghavnadzor, Marmarik R. (site 13), 26.viii.2015, 1 ♀; Kotayk: Hrazdan Sewage Treatment Plant, Hrazdan R. (site 7), 27.viii.2015, 1 ♂; Kotayk: between Marmarik and Aghavnadzor, tributary of Marmarik R. (site 4), 27.viii.2015, 1 ♀; Tavush: W of Dilijan, Bldan R. (site 28), 28.viii.2015, 1 ♂; Tavush: below Jukhtakvank Monastery, (site 26), 29.viii.2015, 1 ♀; Gegharkunik: Tsovagyugh, nr. Sevan L. (site 3), 29.viii.2015, 1 ♀; Lori: N of Dzoraget, tributary of Pambak R. (site 17), 1.ix.2015, 1 ♂; Lori: Meghvahovit, road H31, small steppe brook (site 16), 2.ix.2015, 3 ♀; Shirak: NE of Musayelyan, tributary of Akhurian R. (site 23), 2.ix.2015, 1 ♂ 1 ♀; Shirak: NW of Amasia, tributary of Akhurian R. (site 24), 2.ix.2015, 1 ♂ 1 ♀.

####### Distribution.

Europe; Georgia, Armenia, Azerbaijan, Turkey, Israel, ?Iran; Central Asia; West Siberia.

###### Erioptera (Mesocyphona) bivittata

Taxon classificationAnimaliaDipteraLimoniidae

(Loew, 1873)

####### Material examined.

Lori: N of Pushkin, tributary of Dzoraget R. (site 18), 3.ix.2015, 1 ♂.

####### Distribution.

Europe; Azerbaijan, Turkey, Israel, Iran; Central Asia, Mongolia; West Siberia, East Siberia. First record from Armenia.

###### Hoplolabis (Parilisia) iranica

Taxon classificationAnimaliaDipteraLimoniidae

(Alexander, 1973)

####### Material examined.

Lori: Lermontov, tributary of Aghstev R. (site 15), 1.ix.2015, 4 ♂ 2 ♀.

####### Distribution.

Russia (North Caucasus); Georgia, Azerbaijan, Iran. First record from Armenia.

###### Ilisia maculata

Taxon classificationAnimaliaDipteraLimoniidae

(Meigen, 1804)

####### Material examined.

Gegharkunik: Tsovagyugh, nr. Sevan L. (site 3), 29.viii.2015, 2 ♂; Lori: Lermontov, tributary of Aghstev R. (site 15), 1.ix.2015, 1 ♀; Shirak: E of Torosgyugh, tributary of Akhurian R. (site 22), 3.ix.2015, 3 ♂ 1 ♀.

####### Distribution.

Europe; Georgia, Armenia, Azerbaijan, Turkey, Iran.

###### Molophilus (Molophilus) lackschewitzianus habetatus

Taxon classificationAnimaliaDipteraLimoniidae

Savchenko, 1976

####### Material examined.

Tavush: E of Haghartsin, tributary of Aghstev R. (site 29), 29.viii.2015, 1 ♂.

####### Distribution.

Russia (North Caucasus); Georgia, Armenia.

####### Remark.

Possibly a valid species.

###### Molophilus (Molophilus) obscurus

Taxon classificationAnimaliaDipteraLimoniidae

(Meigen, 1818)

####### Material examined.

Shirak: NE of Musayelyan, tributary of Akhurian R. (site 23), 2.ix.2015, 2 ♂.

####### Distribution.

Europe; Morocco; Georgia, Armenia, Turkey, Cyprus, Lebanon, Israel.

###### Molophilus (Molophilus) ochraceus

Taxon classificationAnimaliaDipteraLimoniidae

(Meigen, 1818)

####### Material examined.

Lori: N of Dzoraget, tributary of Pambak R. (site 17), 1.ix.2015, 2 ♂.

####### Distribution.

Europe; Georgia, Azerbaijan, Turkey. First record from Armenia.

###### Molophilus (Molophilus) propinquus

Taxon classificationAnimaliaDipteraLimoniidae

(Egger, 1863)

####### Material examined.

Tavush: N of Gosh, Getik R. (site 31), 4.ix.2015, 10 ♂ 3 ♀.

####### Distribution.

Europe; Morocco; Georgia, Turkey; West Siberia, East Siberia, Far East of Russia. First record from Armenia.

###### Molophilus (Molophilus) pleuralis

Taxon classificationAnimaliaDipteraLimoniidae

de Meijere, 1920

####### Material examined.

Kotayk: NW of Artavaz, Marmarik R. (site 6), 26.viii.2015, 3 ♂; Shirak: NW of Amasia, tributary of Akhurian R. (site 24), 2.ix.2015, 2 ♂.

####### Distribution.

Europe; Georgia, Azerbaijan, Turkey, Cyprus, Israel, Iran; Central Asia; as far east as Far East of Russia. First records from Armenia.

###### Molophilus (Molophilus) stroblianus decoloratus

Taxon classificationAnimaliaDipteraLimoniidae

Savchenko, 1978

####### Material examined.

Kotayk: Meghradzor, below “Gold Mine”, tributary of Marmarik R. (site 9), 27.viii.2015, 2 ♂ 2 ♀; Tavush: W of Dilijan, Bldan R. (site 28), 28.viii.2015, 3 ♂ 1 ♀; Tavush: below Jukhtakvank Monastery, (site 26), 29.viii.2015, 1 ♀; Lori: Lermontov, tributary of Aghstev R. (site 15), 1.ix.2015, 4 ♂ 4 ♀.

####### Distribution.

Ukraine, Russia (North Caucasus); Georgia, Armenia, Azerbaijan. First records since original description.

####### Remark.

Possibly a valid species.

###### Ormosia (Ormosia) cuspidata

Taxon classificationAnimaliaDipteraLimoniidae

Savchenko, 1973

####### Material examined.

Lori: N of Dzoraget, tributary of Pambak R. (site 17), 1.ix.2015, 1 ♂.

####### Distribution.

?European Russia (southeast); Georgia. First record from Armenia.

###### Ormosia (Ormosia) hederae

Taxon classificationAnimaliaDipteraLimoniidae

(Curtis, 1835)

####### Material examined.

Gegharkunik: Tsovagyugh, nr. Sevan L. (site 3), 29.viii.2015, 1 ♀; Lori: Lermontov, tributary of Aghstev R. (site 15), 1.ix.2015, 2 ♀; Shirak: NW of Amasia, tributary of Akhurian R. (site 24), 2.ix.2015, 1 ♂; Shirak: E of Torosgyugh, tributary of Akhurian R. (site 22), 3.ix.2015, 1 ♂.

####### Distribution.

Europe; Georgia, Azerbaijan, Turkey; Tajikistan. First records from Armenia.

###### Rhabdomastix (Rhabdomastix) filata

Taxon classificationAnimaliaDipteraLimoniidae

Starý, 2004

####### Material examined.

Lori: Lermontov, tributary of Aghstev R. (site 15), 1.ix.2015, 1 ♂ 1 ♀.

####### Distribution.

Bulgaria, Greece, Russia (North Caucasus); Georgia, Armenia, Turkey, Lebanon. First record since original description.

###### Symplecta (Symplecta) hybrida

Taxon classificationAnimaliaDipteraLimoniidae

(Meigen, 1804)

####### Material examined.

Shirak: NW of Amasia, tributary of Akhurian R. (site 24), 2.ix.2015, 1 ♀.

####### Distribution.

Nearctic (Canada, USA, Greenland); widespread in Palaearctic, including Europe; North Africa; Georgia, Armenia, Azerbaijan, Turkey, Lebanon, Israel, Iran; Central Asia, Mongolia; as far east as North Korea, Japan and China; Oriental (India, Nepal, Pakistan).

##### Subfamily Limoniinae

###### Achyrolimonia decemmaculata

Taxon classificationAnimaliaDipteraLimoniidae

(Loew, 1873)

####### Material examined.

Tavush: W of Dilijan, Bldan R. (site 28), 28.viii.2015, 1 ♀; Tavush: NW of Teghut, tributary of Aghstev R. (site 32), 29.viii.2015, 1 ♀.

####### Distribution.

Europe; Georgia, Armenia, Azerbaijan, Iran.

###### Antocha (Antocha) vitripennis

Taxon classificationAnimaliaDipteraLimoniidae

(Meigen, 1830)

####### Material examined.

Kotayk: between Marmarik and Aghavnadzor, tributary of Marmarik R. (site 4), 27.viii.2015, 1 ♂; Tavush: W of Dilijan, Bldan R. (site 28), 28.viii.2015, 1 ♀ 1 ♂.

####### Distribution.

Europe; Turkey, Israel; Afghanistan. First records from Armenia and Transcaucasia

###### Dicranomyia (Dicranomyia) circassica

Taxon classificationAnimaliaDipteraLimoniidae

Lackschewitz, 1941

####### Material examined.

Tavush: car park on road M4, tributary of Aghstev R. (site 27), 4.ix.2015, 1 ♂.

####### Distribution.

So far only known from North Caucasus and Georgia. First record from Armenia.

###### Dicranomyia (Dicranomyia) didyma

Taxon classificationAnimaliaDipteraLimoniidae

(Meigen, 1804)

####### Material examined.

Kotayk: E of Hankavan, Marmarik R. (site 5), 26.viii.2015, 1 ♀; Kotayk: N of Solak, Hrazdan R. (site 11), 27.viii.2015, 1 ♂; Ararat: nr. Lanjazat, Azat R. (site 2), 31.viii.2015, 1 ♀; Shirak: NE of Musayelyan, tributary of Akhurian R. (site 23), 2.ix.2015, 1 ♀; Shirak: NW of Amasia, tributary of Akhurian R. (site 24), 2.ix.2015, 1 ♀.

####### Distribution.

Europe; Morocco, Algeria; Georgia, Armenia, Azerbaijan, Turkey, Iran; Afghanistan, Mongolia, ?China.

###### Dicranomyia (Dicranomyia) longipennis

Taxon classificationAnimaliaDipteraLimoniidae

(Schummel, 1829)

####### Material examined.

Kotayk: Meghradzor, behind railway, tributary of Marmarik R. (site 8), 27.viii.2015, 4 ♂, 1 ♀.

####### Distribution.

Nearctic (Canada, USA); widespread in Palaearctic, including Europe; Georgia, Azerbaijan, ?Syria, Iran; Central Asia, Mongolia; as far east as Far East of Russia and Japan; Oriental (India). First record from Armenia.

###### Dicranomyia (Dicranomyia) melanantha

Taxon classificationAnimaliaDipteraLimoniidae

Savchenko, 1984

####### Material examined.

Kotayk: between Marmarik and Aghavnadzor, tributary of Marmarik R. (site 4), 27.viii.2015, 1 ♀; Lori: Lermontov, tributary of Aghstev R. (site 15), 1.ix.2015, 2 ♂ 1 ♀; Lori: Meghvahovit, road H31, small steppe brook (site 16), 2.ix.2015, 6 ♂ 1 ♀; Shirak: NE of Musayelyan, tributary of Akhurian R. (site 23), 2.ix.2015, 1 ♂; Tavush: car park on road M4, tributary of Aghstev R. (site 27), 4.ix.2015, 1 ♂.

####### Distribution.

France (Corsica), Russia (North Caucasus); Georgia, Azerbaijan, ?Lebanon. First records from Armenia.

###### Dicranomyia (Dicranomyia) modesta

Taxon classificationAnimaliaDipteraLimoniidae

(Meigen, 1818)

####### Material examined.

Gegharkunik: Tsovagyugh, nr. Sevan L. (site 3), 29.viii.2015, 1 ♂; Lori: NE of Geghasar, Pambak R. (site 19), 3.ix.2015, 1♀; Tavush: N of Gosh, Getik R. (site 31), 4.ix.2015, 1 ♂.

####### Distribution.

Nearctic (Canada, USA, Greenland); widespread in Palaearctic, including Europe; Georgia, Armenia, Azerbaijan, Turkey, Iran; Central Asia, Mongolia; as far east as Far East of Russia and Japan.

###### Dicranomyia (Dicranomyia) pallidinota

Taxon classificationAnimaliaDipteraLimoniidae

Starý, 2009

####### Material examined.

Gegharkunik: Tsovagyugh, nr. Sevan L. (site 3), 29.viii.2015, 1 ♂.

####### Distribution.

Bulgaria, Greece, France (Corsica); Lebanon, Syria. First record from Armenia and Transcaucasia; first record since original description.

###### Dicranomyia (Dicranomyia) pontica

Taxon classificationAnimaliaDipteraLimoniidae

Lackschewitz, 1941

####### Material examined.

Tavush: W of Dilijan, Bldan R. (site 28), 28.viii.2015, 1 ♂; Lori: W of Vahagnadzor, Zamanlu R. (site 14), 1.ix.2015, 2 ♂ 1 ♀.

####### Distribution.

So far only known from North Caucasus and Georgia. First records from Armenia.

###### Dicranomyia (Numantia) fusca

Taxon classificationAnimaliaDipteraLimoniidae

(Meigen, 1804)

####### Material examined.

Kotayk: near Artavaz, Marmarik R. (site 10), 27.viii.2015, 1 ♂; Kotayk: between Marmarik and Aghavnadzor, tributary of Marmarik R. (site 4), 27.viii.2015, 2 ♂; Lori: road H23 to Pushkin Pass, small brook (site 20), 3.ix.2015, 1 ♂.

####### Distribution.

Nearctic (Canada, USA); Europe; Georgia, Azerbaijan, Turkey, Iran; Far East of Russia, Japan. First records from Armenia.

###### Dicranoptycha livescens

Taxon classificationAnimaliaDipteraLimoniidae

Loew, 1871

####### Material examined.

Tavush: E of Matosavank Monastery (site 30), 29.viii.2015, 1 ♀; Tavush: E of Haghartsin, tributary of Aghstev R. (site 29), 29.viii.2015, 1 ♂.

####### Distribution.

Europe, except for northern countries. First records from Armenia and Transcaucasia.

###### Limonia hercegovinae

Taxon classificationAnimaliaDipteraLimoniidae

(Strobl, 1898)

####### Material examined.

Tavush: W of Dilijan, Bldan R. (site 28), 28.viii.2015, 1 ♂; Tavush: below Jukhtakvank Monastery, (site 26), 29.viii.2015, 1 ♂ 1 ♀; Tavush: NW of Teghut, tributary of Aghstev R. (site 32), 29.viii.2015, 1 ♂; Tavush: E of Haghartsin, tributary of Aghstev R. (site 29), 29.viii.2015, 1 ♂; Lori: W of Vahagnadzor, Zamanlu R. (site 14), 1.ix.2015, 2 ♂; Lori: road H23 to Pushkin Pass, small brook (site 20), 3.ix.2015, 1 ♀.

####### Distribution.

Europe, except for northern countries; Morocco; Azerbaijan, Turkey, Iran. First records from Armenia.

###### Limonia macrostigma

Taxon classificationAnimaliaDipteraLimoniidae

(Schummel, 1829)

####### Material examined.

Lori: Lermontov, tributary of Aghstev R. (site 15), 1.ix.2015, 1 ♂; Shirak: NW of Amasia, tributary of Akhurian R. (site 24), 2.ix.2015, 1 ♂.

####### Distribution.

Europe; Morocco; Georgia, Azerbaijan, Turkey, Cyprus; Central Asia; as far east as Far East of Russia, and ?North Korea; Oriental (Pakistan). First records from Armenia.

###### Limonia stigma

Taxon classificationAnimaliaDipteraLimoniidae

(Meigen, 1818)

####### Material examined.

Gegharkunik: Tsovagyugh, nr. Sevan L. (site 3), 29.viii.2015, 1 ♂; Lori: Lermontov, tributary of Aghstev R. (site 15), 1.ix.2015, 1 ♂.

####### Distribution.

Europe. First records from Armenia and Transcaucasia.

###### Metalimnobia (Metalimnobia) quadrinotata

Taxon classificationAnimaliaDipteraLimoniidae

(Meigen, 1818)

####### Material examined.

Kotayk: E of Hankavan, Marmarik R. (site 5), 26.viii.2015, 1 ♂.

####### Distribution.

Europe; Kirghizia, Mongolia; West Siberia, East Siberia, Far East of Russia. First record from Armenia and Transcaucasia.

###### Rhipidia (Rhipidia) maculata

Taxon classificationAnimaliaDipteraLimoniidae

Meigen, 1818

####### Material examined.

Gegharkunik: Tsovagyugh, nr. Sevan L. (site 3), 29.viii.2015, 1 ♂.

####### Distribution.

Nearctic (Canada, USA); widespread in Palaearctic, including Europe; Georgia; Mongolia; as far east as Far East of Russia, China, and Japan; Oriental (China). First record from Armenia.

#### Family Pediciidae

##### Dicranota (Dicranota) crassicauda

Taxon classificationAnimaliaDipteraPediciidae

Tjeder, 1972

###### Material examined.

Shirak: Zuygaghbyur, meanders of tributary of Akhurien R. (site 25), 2.ix.2015, 1 ♂; Shirak: E of Torosgyugh, tributary of Akhurian R. (site 22), 3.ix.2015, 1 ♂.

###### Distribution.

Finland, Norway, Sweden; Kazakhstan, Tajikistan. First records from Armenia and Transcaucasia.

##### Dicranota (Paradicranota) landrocki

Taxon classificationAnimaliaDipteraPediciidae

Czižek, 1931

###### Material examined.

Shirak: NW of Amasia, tributary of Akhurian R. (site 24), 2.ix.2015, 1 ♂.

###### Distribution.

Europe, except for northern countries; Morocco; Georgia, Armenia, Azerbaijan, Lebanon; Tajikistan.

##### Dicranota (Paradicranota) subtilis

Taxon classificationAnimaliaDipteraPediciidae

Loew, 1871

###### Material examined.

Tavush: vicinity of Parz L. (site 33), 28.viii.2015, 1 ♂; Tavush: below Jukhtakvank Monastery, (site 26), 29.viii.2015, 1 ♂; Lori: Meghvahovit, road H31, small steppe brook (site 16), 2.ix.2015, 1 ♂.

###### Distribution.

Europe; Georgia, Armenia, Azerbaijan.

##### Pedicia (Amalopis) occulta

Taxon classificationAnimaliaDipteraPediciidae

(Meigen, 1830)

###### Material examined.

Lori: road H23 to Pushkin Pass, small brook (site 20), 3.ix.2015, 1 ♂.

###### Distribution.

Europe; Georgia, Armenia, Azerbaijan, Turkey, Cyprus, Lebanon.

## Discussion

A total of 38 species of Limoniidae and four species of Pediciidae are recorded from Armenia. Of these, 27 species of Limoniidae and one species of Pediciidae represent the first records for Armenia. These are the following: Paradelphomyia (Oxyrhiza) brevifurca, Paradelphomyia (Oxyrhiza) fuscula, Paradelphomyia (Oxyrhiza) senilis, Phylidorea (Phylidorea) ferruginea, Pseudolimnophila (Pseudolimnophila) melanura, Pseudolimnophila (Pseudolimnophila) sepium, *Erioconopa symplectoides*, Erioptera (Mesocyphona) bivittata, Hoplolabis (Parilisia) iranica, Molophilus (Molophilus) ochraceus, Molophilus (Molophilus) pleuralis, Molophilus (Molophilus) propinquus, Ormosia (Ormosia) cuspidata, Ormosia (Ormosia) hederae, Antocha (Antocha) vitripennis, Dicranomyia (Dicranomyia) circassica, Dicranomyia (Dicranomyia) longipennis, Dicranomyia (Dicranomyia) melanantha, Dicranomyia (Dicranomyia) pallidinota, Dicranomyia (Dicranomyia) pontica, Dicranomyia (Numantia) fusca, *Dicranoptycha livescens*, *Limonia hercegovinae*, *Limonia macrostigma*, *Limonia stigma*, Metalimnobia (Metalimnobia) quadrinotata, Rhipidia (Rhipidia) maculata, ﻿and Dicranota (Dicranota) crassicauda. Seven species of Limoniidae and one species of Pediciidae are new to the whole Transcaucasia, viz. Paradelphomyia (Oxyrhiza) fuscula, Pseudolimnophila (Pseudolimnophila) melanura, Antocha (Antocha) vitripennis, Dicranomyia (Dicranomyia) pallidinota, *Dicranoptycha livescens*, *Limonia stigma*, Metalimnobia (Metalimnobia) quadrinotata, ﻿and Dicranota (Dicranota) crassicauda. Four species/subspecies are here recorded for the first time since their original descriptions, viz. Pseudolimnophila (Pseudolimnophila) melanura, Molophilus (Molophilus) stroblianus decoloratus, Rhabdomastix (Rhabdomastix) filata, ﻿and Dicranomyia (Dicranomyia) pallidinota.

Altogether 50 species of Limoniidae and five species of Pediciidae were previously known to occur in Armenia (Oosterbroek 2015). Our records increase the number of Armenian species to 83, 77 species of Limoniidae and six species of Pediciidae.

### Checklist of Limoniidae and Pediciidae of Armenia

species new to Armenia are marked with an asterisk (*)

Limoniidae: Limnophilinae

1. *Afrolimnophila minima* (Savchenko, 1971)

2. Dicranophragma (Brachylimnophila) nemorale (Meigen, 1818)

3. Dicranophragma (Mixolimnomyia) rufulum (Savchenko, 1979)

4. *Eloeophila maculata* (Meigen, 1804)

5. Hexatoma (Cladolipes) haiasana Savchenko, 1972

6. Hexatoma (Hexatoma) gaedii (Meigen, 1830)

7. Limnophila (Limnophila) pictipennis (Meigen, 1818)

8. *Paradelphomyia (Oxyrhiza) brevifurca Savchenko, 1976

9. *Paradelphomyia (Oxyrhiza) fuscula (Loew, 1873)

10. *Paradelphomyia (Oxyrhiza) senilis (Haliday, 1833)

11. *Phylidorea (Phylidorea) ferruginea (Meigen, 1818)

12. Pseudolimnophila (Pseudolimnophila) lucorum (Meigen, 1818)

13. *Pseudolimnophila (Pseudolimnophila) melanura Savchenko, 1984

14. *Pseudolimnophila (Pseudolimnophila) sepium (Verrall, 1886)

Limoniidae Chioneinae

15. Ellipteroides (Ptilostenodes) omissus (Lackschewitz, 1940)

16. *Erioconopa symplectoides (Kuntze, 1914)

17. *Erioconopa trivialis* (Meigen, 1818)

18. Erioptera (Erioptera) fusculenta Edwards, 1938

19. Erioptera (Erioptera) lutea Meigen, 1804

20. *Erioptera (Mesocyphona) bivittata (Loew, 1873)

21. Gonomyia (Gonomyia) basilobata Alexander, 1975

22. Gonomyia (Gonomyia) conoviensis Barnes, 1924

23. Gonomyia (Gonomyia) lucidula de Meijere, 1920

24. Gonomyia (Gonomyia) papposa Savchenko, 1983

25. Hoplolabis (Eurasicesa) amseliana (Nielsen, 1961)

26. *Hoplolabis (Parilisia) iranica (Alexander, 1973)

27. Hoplolabis (Parilisia) yezoana (Alexander, 1924)

28. Idiocera (Idiocera) laterospina (Alexander, 1975)

29. Idiocera (Idiocera) pulchripennis (Loew, 1856)

30. *Ilisia maculata* (Meigen, 1804)

31. Molophilus (Molophilus) lackschewitzianus hebetatus Savchenko, 1976

32. Molophilus (Molophilus) obscurus (Meigen, 1818)

33. *Molophilus (Molophilus) ochraceus (Meigen, 1818)

34. *Molophilus (Molophilus) pleuralis de Meijere, 1920

35. Molophilus (Molophilus) politonigrus Savchenko, 1983

36. *Molophilus (Molophilus) propinquus (Egger, 1863)

37. Molophilus (Molophilus) stroblianus decoloratus Savchenko, 1978

38. Molophilus (Molophilus) urodontus Savchenko, 1978

39. *Ormosia (Ormosia) cuspidata Savchenko, 1973

40. Ormosia (Ormosia) fascipennis (Zetterstedt, 1838)

41. *Ormosia (Ormosia) hederae (Curtis, 1835)

42. Ormosia (Ormosia) longispina Savchenko, 1983

43. *Phyllolabis ghilarovi* Savchenko, 1983

44. Rhabdomastix (Rhabdomastix) eugeni Stary, 2004

45. Rhabdomastix (Rhabdomastix) filata Stary, 2004

46. Symplecta (Psiloconopa) stictica (Meigen, 1818)

47. Symplecta (Symplecta) hybrida (Meigen, 1804)

Limoniidae: Limoniinae

48. *Achyrolimonia decemmaculata* (Loew, 1873)

49. Antocha (Antocha) libanotica Lackschewitz, 1940

50. *Antocha (Antocha) vitripennis (Meigen, 1830)

51. *Dicranomyia (Dicranomyia) circassica Lackschewitz, 1941

52. Dicranomyia (Dicranomyia) didyma (Meigen, 1804)

53. Dicranomyia (Dicranomyia) chorea (Meigen, 1818)

54. *Dicranomyia (Dicranomyia) longipennis (Schummel, 1829)

55. Dicranomyia (Dicranomyia) lucida de Meijere, 1918

56. *Dicranomyia (Dicranomyia) melanantha Savchenko, 1984

57. Dicranomyia (Dicranomyia) modesta (Meigen, 1818)

58. *Dicranomyia (Dicranomyia) pallidinota Starý, 2009

59. *Dicranomyia (Dicranomyia) pontica Lackschewitz, 1941

60. Dicranomyia (Glochina) transsilvanica Lackschewitz, 1928

61. Dicranomyia (Melanolimonia) caledonica Edwards, 1926

62. Dicranomyia (Melanolimonia) morio (Fabricius, 1787)

63. *Dicranomyia (Numantia) fusca (Meigen, 1804)

64. *Dicranoptycha fuscescens* (Schummel, 1829)

65. *Dicranoptycha livescens Loew, 1871

66. *Dicranoptycha recurvispina* Savchenko, 1974

67. *Limonia caucasica* Lackschewitz, 1940

68. *Limonia eos* Stary & Savchenko, 1976

69. *Limonia flavipes* (Fabricius, 1787)

70. *Limonia hercegovinae (Strobl, 1898)

71. *Limonia macrostigma (Schummel, 1829)

72. *Limonia nubeculosa* Meigen, 1804

73. *Limonia stigma (Meigen, 1818)

74. *Limonia subaequalis* Savchenko, 1979

75. Metalimnobia (Metalimnobia) quadrimaculata (Linnaeus, 1760)

76. *Metalimnobia (Metalimnobia) quadrinotata (Meigen, 1818)

77. *Rhipidia (Rhipidia) maculata Meigen, 1818

Pediciidae

78. *Dicranota (Dicranota) crassicauda Tjeder, 1972

79. Dicranota (Ludicia) iranensis (Alexander, 1975)

80. Dicranota (Paradicranota) landrocki Czižek, 1931

81. Dicranota (Paradicranota) subtilis Loew, 1871

82. Pedicia (Amalopis) occulta (Meigen, 1830)

83. Tricyphona (Tricyphona) immaculata (Meigen, 1804)

## Supplementary Material

XML Treatment for Paradelphomyia (Oxyrhiza) brevifurca

XML Treatment for Paradelphomyia (Oxyrhiza) fuscula

XML Treatment for Paradelphomyia (Oxyrhiza) senilis

XML Treatment for Phylidorea (Phylidorea) ferruginea

XML Treatment for Pseudolimnophila (Pseudolimnophila) melanura

XML Treatment for Pseudolimnophila (Pseudolimnophila) sepium

XML Treatment for Erioconopa symplectoides

XML Treatment for Erioptera (Erioptera) fusculenta

XML Treatment for Erioptera (Erioptera) lutea

XML Treatment for Erioptera (Mesocyphona) bivittata

XML Treatment for Hoplolabis (Parilisia) iranica

XML Treatment for Ilisia maculata

XML Treatment for Molophilus (Molophilus) lackschewitzianus habetatus

XML Treatment for Molophilus (Molophilus) obscurus

XML Treatment for Molophilus (Molophilus) ochraceus

XML Treatment for Molophilus (Molophilus) propinquus

XML Treatment for Molophilus (Molophilus) pleuralis

XML Treatment for Molophilus (Molophilus) stroblianus decoloratus

XML Treatment for Ormosia (Ormosia) cuspidata

XML Treatment for Ormosia (Ormosia) hederae

XML Treatment for Rhabdomastix (Rhabdomastix) filata

XML Treatment for Symplecta (Symplecta) hybrida

XML Treatment for Achyrolimonia decemmaculata

XML Treatment for Antocha (Antocha) vitripennis

XML Treatment for Dicranomyia (Dicranomyia) circassica

XML Treatment for Dicranomyia (Dicranomyia) didyma

XML Treatment for Dicranomyia (Dicranomyia) longipennis

XML Treatment for Dicranomyia (Dicranomyia) melanantha

XML Treatment for Dicranomyia (Dicranomyia) modesta

XML Treatment for Dicranomyia (Dicranomyia) pallidinota

XML Treatment for Dicranomyia (Dicranomyia) pontica

XML Treatment for Dicranomyia (Numantia) fusca

XML Treatment for Dicranoptycha livescens

XML Treatment for Limonia hercegovinae

XML Treatment for Limonia macrostigma

XML Treatment for Limonia stigma

XML Treatment for Metalimnobia (Metalimnobia) quadrinotata

XML Treatment for Rhipidia (Rhipidia) maculata

XML Treatment for Dicranota (Dicranota) crassicauda

XML Treatment for Dicranota (Paradicranota) landrocki

XML Treatment for Dicranota (Paradicranota) subtilis

XML Treatment for Pedicia (Amalopis) occulta
